# Analysis of Dielectric Parameters of Fe_2_O_3_-Doped Polyvinylidene Fluoride/Poly(methyl methacrylate) Blend Composites

**DOI:** 10.3390/molecules28155722

**Published:** 2023-07-28

**Authors:** Minal Bafna, Farah Deeba, Ankit K. Gupta, Kriti Shrivastava, Vaibhav Kulshrestha, Ankur Jain

**Affiliations:** 1 Department of Physics, Agrawal P. G. College, Jaipur 302003, India; 2 Department of Physics, S. S. Jain Subodh P. G. College, Jaipur 302004, India; 3 School of Applied Sciences, Suresh Gyan Vihar University, Jaipur 302017, India; 4 Center for Renewable Energy and Storage, Suresh Gyan Vihar University, Jaipur 302017, India; 5 CSIR-Central Salt and Marine Chemicals Research Institute, Bhavnagar 364002, India; 6 Natural Science Centre for Basic Research & Development, Hiroshima University, Higashihiroshima 739-8530, Japan

**Keywords:** thin film, PVDF/PMMA polymer blends, ferric oxide, electrical conductivity, complex dielectric function, electric modulus

## Abstract

In this paper, we report the effect of metal oxide (Fe_2_O_3_) loading in different weight ratios (0.5%, 1%, 2%, and 4%) on the structural and electrical parameters, viz., the complex dielectric constant, electric modulus spectra, and the AC conductivity, of polymeric composites of PVDF/PMMA (30/70 weight ratio) blend. The structural and geometric measurements have been analyzed with the help of peak location, peak intensity, and peak shape obtained from XRD as well as from FTIR spectra. The electrical properties have been investigated using an impedance analyzer in the frequency range 100 Hz to 1 MHz. The real parts of the complex permittivity and the dielectric loss tangent of these materials are found to be frequency independent in the range from 20 KHz to 1 MHz, but they increase with the increase in the concentration of nano-Fe_2_O_3_. The conductivity also increases with an increased loading of Fe_2_O_3_ in PVDF/PMMA polymer blends. The electric modulus spectra were used to analyze the relaxation processes associated with the Maxwell–Wagner–Sillars mechanism and chain segmental motion in the polymer mix.

## 1. Introduction

Polymer nano composites (PNC) are basically a polymer matrix containing small amounts of inorganic filler homogeneously dispersed by means of a state- of-the-art process. They are gaining considerable interest from researchers due to their numerous uses in current cutting-edge technologies and devices in the rapidly expanding and changing field of science [1,2,3,4,5,6]. PNCs have the capacity to incorporate the advantageous characteristics of both nanofillers and polymer mixtures. In comparison to pure polymers, doped polymer nanocomposites might offer a wide range of industrial applications. Numerous nano fillers can significantly enhance the thermophysical properties of polymer nanocomposites, enabling a wide range of commercial applications in industries like electronics, optics, energy, sensors, and healthcare [7,8,9,10,11]. Several studies have indicated [12,13,14,15,16,17,18,19,20,21,22,23,24,25,26,27,28,29,30,31] improved composite properties as a result of doping polymers with various nano fillers. The incorporation of carbon soot into polymers enhances their thermal conductivity and mechanical properties, such as tensile strength and hardness, making them suitable for applications where efficient heat dissipation is required. The doping of potassium- based ternary oxides (KMnO_4_ and K_2_CrO_4_); tin salts (SnCl_2_and SnO_2_); and ZnO in polymers has been shown to alter optical [5,7,15,16,28] and electrical [3,11,16,22,25,27] properties, thus enabling them to be used as significant EM shielding devices, coatings, textiles, and biomedical devices. FeCl_3_ [19,30] doping was found suitable for enhancing optical, magnetic, and electrical properties, making it suitable for magnetic resonance imaging (MRI), magnetic sensors, and electromagnetic devices.

When insulating polymers and conducting fillers are combined, they exhibit intriguing electrical behavior [1,4,7,9,12,14,18,21,22,23,24,25,26,27,31,32,33,34]. The interaction between the insulating polymer matrix and the conducting filler leads to the formation of powerful interfaces that actively and passively influence the dielectric behavior of the composite. Upon dispersion of the filler into the polymer matrix, there are changes in the local charge distribution and morphology at the interfaces between the two materials. This alteration affects the density and energy depths of the trap sites, which in turn influences the charge mobility and resulting dielectric properties of the composite. At lower concentrations, the filler particles are sufficiently spaced apart, preventing the creation of a conductive path. In this regime, the electrical behavior of the composite is primarily influenced by interfacial polarization occurring at the interface. Thus, the electrical behavior remains the same as that of the original host material. However, when the filler is added in adequate amounts, a percolation path is formed, connecting the filler particles. This percolation path allows for charge transport through the surface of the filler particles, leading to a change in the charge storage capacity of the composite [7,18,31].

To analyze the electrical behavior, two important contrasting electrical parameters need to be determined:

1. The capacitive behavior of the composite described by complex electrical permittivity (ε_r_), which quantifies the ability of the material to store electrical energy in an electric field;
2. The conductive behavior of the composite material characterized by the electrical conductivity (σ), which represents the ability of the material to conduct electrical current.

Understanding the electrical behavior of the composite depends on how these electrical properties are affected by the nature, quantity, and addition technique of the fillers. For the design and development of specific materials for dielectric applications in microelectronics, this understanding is crucial. It is feasible to design composites with the appropriate electrical properties for particular applications in the realm of microelectronics by customizing the filler type, concentration, and method.

In this present study, we aimed to investigate the effect of doping Fe_2_O_3_ nanofillers at varying concentrations (0%, 0.5% 1%, 2%, and 4% by weight) in the PVDF/PMMA composite blend with a ratio of 3:7. In our previous research, we observed that blending PMMA with PVDF enhances the β-phase of PVDF, resulting in significant dielectric permittivity and extremely low dielectric losses in the blend, making it a suitable host polymer matrix [24,32]. In general, oxides have been attractive materials for enhancing the electrical properties of polymer composites [32,33,34,35,36,37,38,39,40,41]. For instance, SnO_2_ addition enhances properties such as dielectric permittivity and AC electrical conductivity of poly vinyl pyrrolidone [33]. The Fe_2_O_3_ nanoparticles have been shown to exhibit semiconducting behavior, particularly in their hematite form (α-Fe_2_O_3_), making them useful in electronic devices, chemical sensors, and energy storage systems. According to a comprehensive review of the literature, adding ferric oxide nanoparticles to polymers alters the material’s structural, optical, ferromagnetic, or superparamagnetic behavior noticeably [38,39,40,41]. In a report by Bashir et al., it was shown that the interaction between Fe_2_O_3_ and polyaniline enhanced conductivity to a great extent [40]. In general, it is believed that the increment in complex permittivity with increasing Fe_2_O_3_ nanofiller can be attributed to the polarization process due to the enhanced conductivity and interfacial polarization in the composite and the hopping exchange of charges between localized states. A further reason for the influence on the relative complex permittivity of the nanocomposites is the particle size of the Fe_2_O_3_ nanofiller, since the nano size had a high specific surface area that enabled good contact between the particles and the matrix. The increment in ε′ and ε″ at low frequency can be attributed to the dominant role of dipolar and interfacial polarization. However, the increment at high frequencies can be attributed to the electronic and ionic polarization of the system. Collectively, α-Fe_2_O_3_ is a low-cost ceramic material with dielectric constant *ε*′= ε_r_ = 30; conductivity σ = 10^−5^ to 10^−6^ S-cm^−1^; and band gap = 2.1 eV. With these fascinating properties, the incorporation of α-Fe_2_O_3_ in PVDF/PMMA blends is expected to facilitate the following:

- Charge storage in the polymer blend resulting in the enhancement of the dielectric constant;
- Fe_2_O_3_ nanoparticles acting as conductive fillers, providing additional pathways for electron transport resulting in an increase in overall conductivity;
- An expected enhancement of dielectric losses, as conductivity would be increasing;
- An alteration in the relaxation timescales and the distribution of relaxation processes, resulting in changes in impedance and frequency-dependent electrical behavior.

We expect these resulting polymeric composites to attain the advantage of being light weight and low cost, with ease of mass processibility with a required moderate dielectric constant in the range 10–30 and enhanced conductivity providing advantages in applications that require efficient charge transport or electrical connectivity, such as electronic devices, sensors, or conductive coatings. There have been no studies on the electric properties of Fe_2_O_3_-doped PVDF/PMMA blends so far. Therefore, in this paper, we present the variation in dielectric parameters such as the real part (ε’) and imaginary part (ε″) of the dielectric permittivity, as well as the real part (M′) and imaginary part (M″) of the electrical modulus and the AC conductivity (σ_ac_) of Fe_2_O_3_-doped PVDF/PMMA blends analyzed in the frequency range of 10^2^–10^6^ Hz at room temperature. By systematically studying the electrical behavior of these composites, we can gain insights into the impact of Fe_2_O_3_ doping on their dielectric properties. Studies of XRD and FTIR spectra provide insights into the structural features of the produced PNCs.

## 2. Results and Discussion

### 2.1. XRD Analysis of Prepared Polymer Blend Nanocomposite PNC Films

The X-ray diffraction patterns of the prepared samples were recorded in the angular range 5–90° using the PANalytical X’Pert Pro system with radiation (λ = 1.5408 Å). The value of the Bragg angle (2θ) obtained on XRD characterization is used to identify the values of basal distance (d) using Bragg’s equation

2dsinθ = nλ (1)

**Figure 1:**
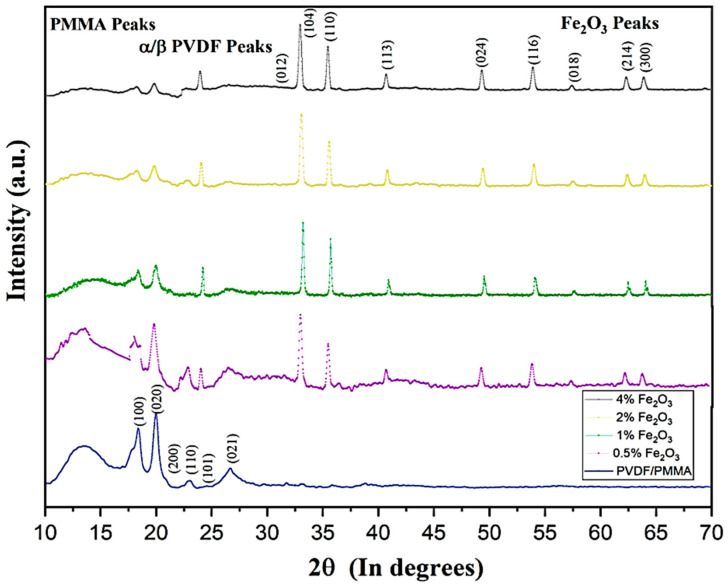
XRD stack representation of Fe_2_O_3_-doped polymer composites PVDF/PMMA (by wt% ratio 30/70).

Figure 1 represents the X-ray diffraction pattern of PVDF/PMMA blend polymers doped with different amounts of Fe_2_O_3_ (0, 0.5%, 1%, 2%, and 4wt %). The PVDF/PMMA matrix shows a semi-crystalline structure in the XRD pattern, with the arrangement of molecules allowing both the α and β phases of PVDF to coexist. PMMA is found to exist primarily in an amorphous state, with a broad diffraction peak in between 2θ = 10° to 18° exactly localized at 2θ = 13.59° in all curves. The intensity of this broad peak decreases with an increase in Fe_2_O_3_ content, as seen in curves (b–e) of Figure 1. The PVDF diffraction peaks with different alpha phases, seen at 2θ = 18.43°, 20.015°, 22.93°, and 26.67°, correspond to (1 0 0), (0 2 0), (1 1 0), and (0 2 1) planes, and the strong peaks of β-phase observed at 2θ = 20.06° and 2θ = 23.16° correspond to the planes (200) and (101).These are in agreement with those reported in our earlier work as well as those reported by Dhatarwal et al., 2020 [12,24,32,33]. Iron oxides commonly exist in either hematite (α-Fe_2_O_3_) or maghemite (γ-Fe_2_O_3_) crystal structures; however, here, only one significantly rhombohedral hematite phase is visible in the XRD measurements for hematite powder, i.e., α-Fe_2_O_3_. The curves (b–e) of Figure 1 show an increase in the relative intensity of the diffraction peaks of Fe_2_O_3_ as the content of filler is increased in the nanocomposite. The sharp hematite (α-Fe_2_O_3_) peaks, alongside the host system’s peaks, at 33.38°, 35.69°, 40.89°, 49.48° 54.14°, 57.48°, 61.72°, and 64.77° correspond to (104), (110), (113), (024) (116), (018), (214), and (300) planes, respectively. Here, the indexing of hkl planes of various peaks was carried out for pristine hematite (α-Fe_2_O_3_) JCPDS card no. S 13-534 [38,39,40,41]. Further, we can be assured that the α-Fe_2_O_3_ powder is free of impurities, as can be concluded from the absence of any residual peak in the XRD spectrum.

The variation in crystallite size L of the nanocomposites as a function of Fe_2_O_3_ concentration was estimated by means of Scherrer’s formula:

(2) $$L=\frac{k\lambda }{\beta_{L\;}cos\;\theta }$$

The structural parameters for prepared nanocomposites are listed in Table 1. 

**Figure 2:**
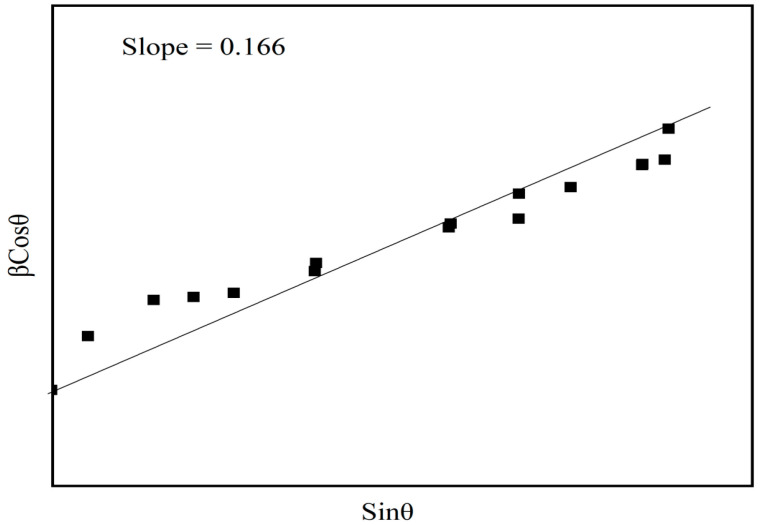
Williamson–Hall plot for PVDF/PMMA (30/70 wt%) with Fe_2_O_3_-doped PNC films.

To confirm the particle size, the size and strain broadening were also calculated using the W–H (Williamson–Hall) formula:

(3) $$\beta_{e}=sC_{\epsilon }tan\;\theta$$

where the values of strain (C) and size (kλ/L) are obtained from the slope and intercept of the W–H plot (βcos θ versus sin θ). The slope and intercept obtained are 0.166 and ~24.12 nm, respectively from Figure 2. Here, the positive value of the slope represents the strain of the Fe_2_O_3_ loaded polymer blend film, which shows the polymer blend film to be of high tensile strength. 

### 2.2. FTIR Spectra and Polymer Blend—Nanofiller Interaction

**Figure 3:**
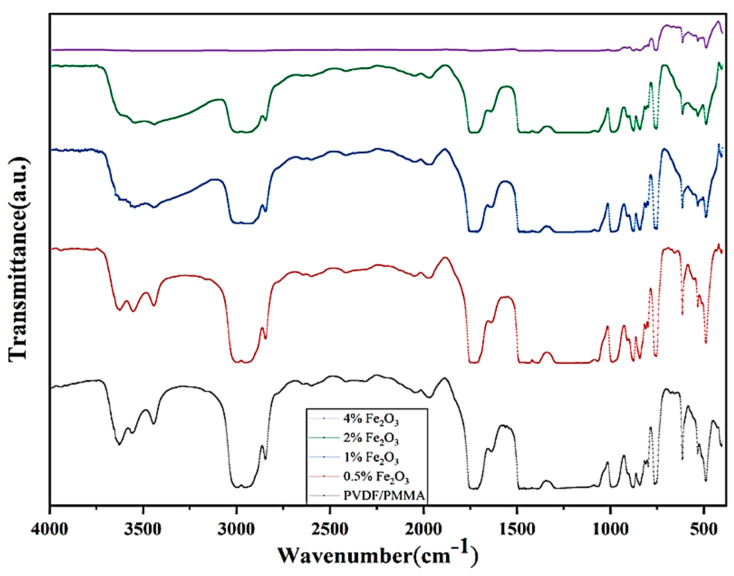
FTIR spectra for Fe_2_O_3_ doped PVDF/PMMA films.

Figure 3 depicts the FTIR spectra in the frequency range 500–4000 cm^−1^, correlating the chemical structure of the PVDF/PMMA blend and Fe_2_O_3_ blend nanocomposites. The FTIR spectrum of the pure polymer blend is in close agreement with one reported earlier [38,39,40,41]. It is clear from the analyzed FTIR spectra that each nano filler -doped PNC film displays the same behavior as that of the pure PB films. A little variation in the intensities of a few of the peaks at lower wave numbers has been observed as compared to the peaks at higher wave numbers (in cm^−1^), which are flattened as the concentration of Fe_2_O_3_ increases. The FTIR patterns showed considerable alterations in several peak intensities and in the shapes of a few bands without any significant deviation in the peak position. This implies no strong chemical interaction between the metal oxide nano particles and the polymer blend matrix. It may be concluded that metal oxide nano particles are basically embedded within the voids between polymer blend chains without affecting the material structure as a physical confinement to the polymer chains only. This leads to slight alterations in the polymer chain packing and the crystalline structure, as is also supported by XRD.

### 2.3. Variation of Dielectric Parameters with Frequency and Composition

When conducting filler is dispersed into a non-conducting polymer matrix, the distribution and mobility of the charge carriers are affected at their interface. At smaller concentrations of filler, interfacial polarization occurs, and capacitive behavior predominates in the system, but when an adequate amount of filler is added so that the particle distance decreases and the filler particles connect to form a conductive path, it facilitates easy charge transport. Such behavior can be analyzed from the values of complex electrical permittivity (ε_r_) and electrical conductivity (σ), which show the capacitive behavior and the conductive behavior of the composite material, respectively. It is crucial to comprehend how these electrical properties depend on the type and amount of filler and the method of filler addition when designing and developing innovative materials for dielectric applications in microelectronics. The Wayne Kerr 6500B impedance analyzer was utilized to measure the impedance parameters, viz., resistance R, capacitance C, and dissipation factor (D = tan δ); using these, the variation of dielectric parameters (ε’ and ε” and tan δ) with frequency (0.1 kHz–1 MHz) and composition (0.5%, 1%, 2%, 4% by wt. of Fe_2_O_3_) was studied and is presented here in this section for the fabricated samples of Fe_2_O_3_-doped PVDF/PMMA blend composites.

#### 2.3.1. Variation in Dielectric Constant

The dielectric constant (ε′), or the real part of the complex electric permittivity (ε_r_ = ε′ + i ε″), is a measure of the charge retention capacity of a medium and depends upon the polarization of the molecules of the material. The greater the polarizability of the molecules, the higher the dielectric constant. It is calculated using the well-known relation for capacitance

ε′ = C t/(ε_0_A) (4)

**Figure 4:**
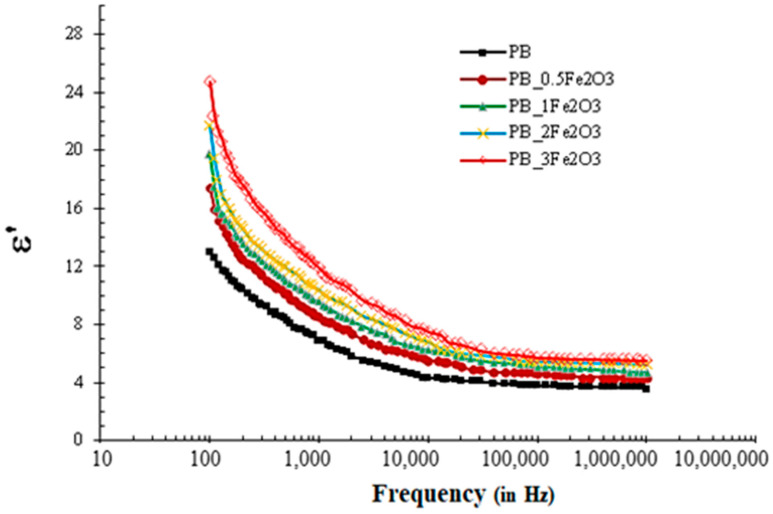
Variation of ε’ with the frequency of PMMA/PVDF-Fe_2_O_3_ films.

Here, the values of capacitance C over the given frequency range were obtained using an impedance analyzer and assuming the thickness t = 10^−4^ m, the surface area of sample A = (10^−2^ m × 10^−2^ m), and ε_0_ = 8.854 × 10^−12^ F/m. The curves for ε′ obtained using this method are depicted in Figure 4 for all the samples. 

Figure 4 reveals the ε′ values of Fe_2_O_3_-doped polymer blend films to be higher than those of the pristine PVDF/PMMA (30/70 wt%) blended films in the entire frequency range. When a small amount of filler (below 3 wt%) is incorporated into the polymer matrix, the nanofillers act as capacitor electrodes, and some microcapacitance structures are formed (the Maxwell–Wagner–Sillars (MWS) effect), resulting in a slight increase in the dielectric constant as compared to that of the pure polymer. On increasing the content of filler material, the host polymer becomes trapped as a very thin dielectric insulating layer of polymer, sandwiched between two neighboring nanoparticle layers. When these nanoparticles become close enough to almost contact each other while still remaining isolated and electrically insulated, they form many parallel or serial microcapacitors; this leads to a high dielectric constant. Concentrations of filler above 5% by weight exhibited an abrupt behavior in measured electrical parameters and are hence not included here. 

It can be further observed that the ε’ values of all the prepared films decrease with an increase in frequency throughout the entire frequency range, with a steep decrease in the 100–10^4^ Hz region, and a gradual variation at the frequencies above 10^4^ Hz. The decrease in these values with an increase in frequency is due to the fact that the molecules undergoing polarization are unable to adopt changes due to the oscillations of the external electric field. Periodic and rapid reversals in the electric field make it impossible for dipoles to align along the applied field. As a result, there is no extra charge dispersion along the electric field, and we see a drop in the values of the dielectric constant. At lower frequencies, the dipoles have enough time to align with the electric field, but as the frequency increases, the dipoles are not able to switch on at the same pace as the electric field [22,24,25,27,32,34,36,41].

#### 2.3.2. Variation of Loss Tangent and Dielectric Loss (ε″)

**Figure 5:**
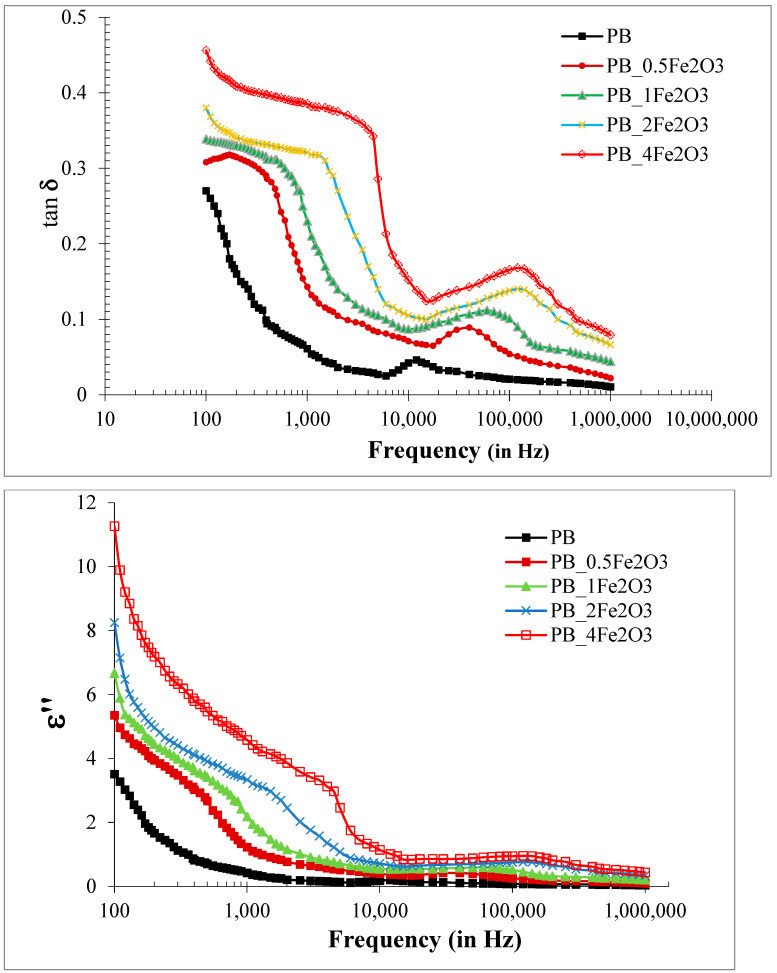
Variation of tan δ (**Top**) and imaginary part of dielectric constant ε″ (**Bottom**) with the frequency for PVDF/PMMA-Fe_2_O_3_ films at room temperature.

The dielectric loss (ε″) of the sample films is calculated using the relation:

(5) ε″= ε′D

where D is the dielectric loss tangent or the dissipation factor (tan δ) spectra obtained for blend composites. Figure 5 depicts the variation of the dielectric loss of PVDF/PMMA-Fe_2_O_3_ composites with an increase in frequency, indicating that ε″obeys the same trend as ε′.

The imaginary part (ε″) of dielectric permittivity, or the dielectric loss, occurs because the molecules undergoing polarization are unable to adopt the same rate of change with which the external electric field oscillates. The time taken by the dipoles to regain their original random orientation, called the relaxation time (τ), is responsible for this. The lagging behind of polarization to the applied electric field is measured by calculating the phase angle between the resistive and reactive components, and it is called the loss tangent or tan δ. Relatively high values of tan δ at low frequencies for the PNC films are observed, which is generally attributed to the leakage current in the polymeric composite samples [22,24,27,34,35,36]. Further, the values of ε″ for the composite films are found to be greater than those for the PVDF/PMMA blend matrix, confirming the increase in energy loss per cycle in the nanocomposites. The addition of Fe_2_O_3_ increases the dielectric constant to the desired extent with a simultaneous undesirable increase in dielectric loss. The gain in the dielectric constant compensates for the gain in losses. The presence of Fe_2_O_3_ nanofiller in the polymer matrix influences the relaxation process and the ability of the material to dissipate energy, and this information is what we analyze from the tan *δ* curve and, consequently, from the ε″ curve. The dielectric relaxation peaks exhibited in their tan *δ* spectra correspond to the mutual local motion of the polymeric chains in the blend, and the broadening of the peaks reflects the overlapping of the IP relaxation process and the merged *αβ* processes. A similar increase in tan δ values on the incorporation of a Fe_2_O_3_ filler in a polymer matrix has also been reported earlier by Bashir and co-researchers [40,41] and in another work, Fe_2_O_3_ has been added into a PVA/PEG blend by Sayed et al. [42], and in the study undertaken on Fe_2_O_3_ in ten kinds of polymer matrices by Kenichi Hayashida [43], a similar trend was observed. Such behavior has been observed even with other metal oxide fillers in different polymers, e.g., when Al_2_O_3_ is inserted in a PVA or PEO-PMMA blend as reported in [14,20], respectively, or when ZnO is inserted in a PVDF/PMMA blend [32], or in research on trends in the use of SnO_2_ in PVP or PEO [33,34].

#### 2.3.3. Variations in Electric Modulus Spectra (M*) Dielectric Parameters with Frequency and Composition

A detailed understanding of electrical behavior is obtained by studying the variation of the electric modulus M∗(ω) = 1/ε∗(ω) = M′ + jM″.

The real part of the electric modulus (M′) and the imaginary part of the electric modulus (M″) are calculated from the evaluated values of ε’ and ε″ as per the relations:

(6) $$M\prime (\omega )=\frac{\varepsilon \prime }{{\left(\varepsilon \prime \prime \right)}^{2}+{\left(\varepsilon \prime \right)}^{2}}\;\;\mathrm{and}\;M\prime \prime (\omega )=\frac{\varepsilon \prime \prime }{{\left(\varepsilon \prime \prime \right)}^{2}+{\left(\varepsilon \prime \right)}^{2}}$$

**Figure 6:**
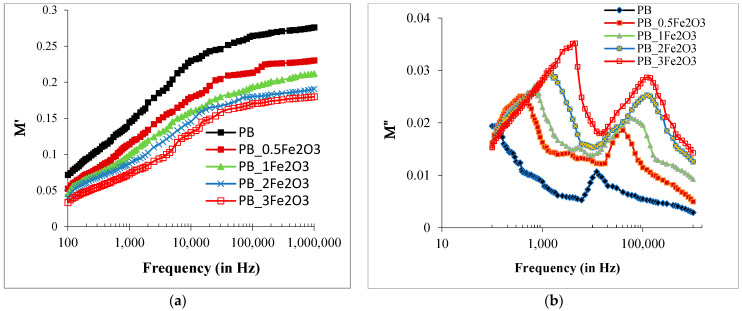
Variation of electric modulus (**a**) real part M′ (**b**) and imaginary part M″ with frequency for PVDF/PMMA-Fe_2_O_3_ films at room temperature.

From Figure 6a, it is found that the M′ values of the PNC films initially have a steeper increase with the increase in frequency from 100 Hz to approximately 10 kHz, whereas in the high-frequency region, these values increase gradually and finally approach the steady state above 100 kHz. The non-zero M′ values in the low-frequency region are evidence that the significant increase in *ε*′ values with the decrease in frequency in the low-frequency range is the bulk property of these PNC materials. The increase in the dielectric permittivity is due to the contribution of the IP effect and is not affected by the EP effect in the *ε*′ values. This type of M′ behavior has also been observed in several polymer composite materials [20,21,22,23,24,25,26,27,28,29,30,31,32,33,34,35,36]. Figure 6b shows that the M″ spectra of the PNC films exhibit the modulus relaxation peaks in the intermediate frequency range. These relaxation peaks of the M″ spectra are relatively sharp as compared to those of the tan δ spectra peaks, and the same characteristic may also be assigned to the polymers’ cooperative chain segmental dynamics in the modulus formalism. On the addition of Fe_2_O_3_ nanoparticles in the PVDF/PMMA blend structure up to 4 wt%, the M″ peak shifts towards the higher- frequency side, which implies that the polymer–nanoparticle interaction reduces the polymer–polymer interaction strength and facilitates the cooperative chain segmental dynamics in the PNC film. The shift in peak towards the higher side can be associated with the reorientation of dipoles, charge redistribution, or other dynamic phenomena occurring at the molecular or microstructural level in the host matrix. As the concentration of filler increases, the peak size is observed to be larger. Larger peak amplitudes indicate higher energy dissipation, suggesting that the composite material exhibits significant electrical loss or charge dissipation behavior. These values are in agreement with the changes observed in the structural properties seen in the XRD and FTIR results.

#### 2.3.4. Electric Conductivity (σ)

The AC electrical conductivity σ_ac_(ω), which depends on the frequency of these PNC films, was measured using relation

**Figure 7:**
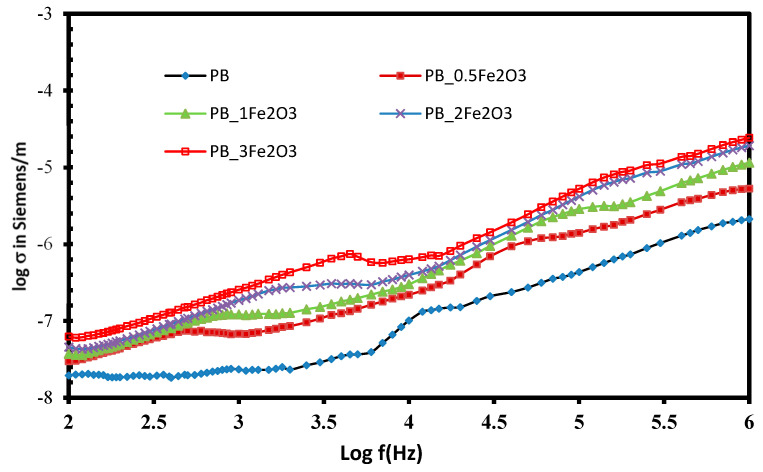
Variation of AC electrical conductivity of Fe_2_O_3_-doped PVDF/PMMA blend films.

σ_ac_(ω) = ωε_0_ε″ (7)

and it has been plotted in Figure 7 as a function of an electric field frequency of 102–106 Hz at room temperature. We can notice that the values are of the order of 10^−7^–10^−8^ S/m at 100 Hz, and this increases with the increase in frequency as well as in filler concentration. The increase with the increase in filler content is obvious due to the increase in charge carriers. Continuous conductive paths of macroscopic length appear in the system, thereby increasing the conductivity with the increase in filler content. The value also rises at higher frequencies due to the short-range intra-well hopping of charge carriers between localized states.

## 3. Materials and Methods

### 3.1. Materials

To ensure reproducibility and allow other researchers to understand and replicate our study’s methodology, we share here the procurement details of the materials used. In this study, for the polymer blend formation, PVDF in powdered form obtained from Sigma Aldrich Chemicals, Pvt. Ltd. (Delhi, India) and PMMA granules from M/s Gharda Chemicals, Bharuch, India were mixed in a ratio of 30:70 by weight. Ferric oxide (Fe_2_O_3_), used as filler material, was obtained from Sigma Aldrich. The solvent used was dichloromethane, which was obtained from Merck India PVT Ltd., Mumbai, India. It had a purity of 99.8%.

### 3.2. Composite Fabrication and Characterization

**Figure 8:**
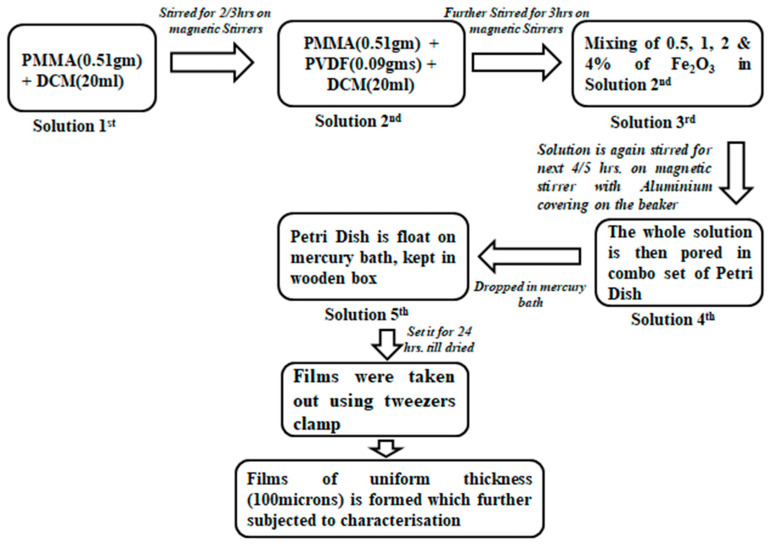
Chart depicting the synthesis of Fe_2_O_3_ PNC films (solution casting method for preparing the PNC films).

The flow chart in Figure 8 below explains the procedure adopted for the fabrication of ferric oxide- doped PVDF/PMMA blend films, which is similar to the steps reported in our previous publications [15,22,24,27,30,32].

**Figure 9:**
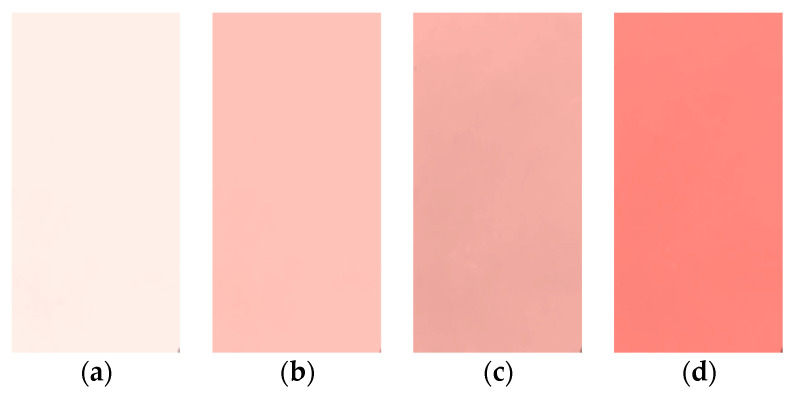
Fe_2_O_3_-doped polymer composite films with different weight ratios: (**a**) 0.5%; (**b**) 1.0%; (**c**) 2.0%; (**d**) 4%.

The obtained films are depicted in Figure 9. These films are clear and with an increasing tinge of reddishness as the content of filler is increased. A number of measurements were performed to obtain information about the uniformity and approximate thickness of the prepared films:

(1) Michelson interferometry was performed, where straight-line fringes were obtained only for films of uniform thickness. The fringes were curved and disturbed for films with non-uniform thickness;
(2) The prepared films were measured at different points using a screw gauge with a least count of 0.01 mm;
(3) The measured thickness was verified by performing a volumetric analysis, where, from the measured values of density and mass and the radius of the petri dish, the thickness of the film was calculated;
(4) The thickness of samples was further measured using optical profilometry. The films were then cut into small pieces with a length–breadth ratio of 1:1 for use in XRD, FTIR, and dielectric spectroscopy to investigate their structural and dielectric properties.

The X-ray diffraction for the structural characterization was performed using the PANalytical X’Pert Pro system, and the W–H (Williamson–Hall) formula was used to determine the size and the strain broadening. The FTIR spectroscopic study was carried out using a Perkin Elmer G-FTIR spectrophotometer. The Wayne Kerr 6500B impedance analyzer was utilized to measure the impedance parameters.

## 4. Conclusions

In studies on complex dielectric permittivity, electrical modulus, and the AC conductivity behavior of PVDF/PMMA films with varying amounts of Fe_2_O_3_, results revealed that the introduction of various weight percentages of metal oxide Fe_2_O_3_ nanofillers in the host polymer blend matrix significantly alters the dielectric polarization, charge transport property, and electrical conduction. Over the entire experimental frequency range 0.1 K Hz to 1 M Hz, it was found that increasing the Fe_2_O_3_ content significantly increases the dielectric constant (ε), AC conductivity (σ_AC_), and loss tangent (tan δ), all of which have higher values than those of the pristine PB film. Further, this dielectric constant decreases with the increase in frequency and increases with the concentration of Fe_2_O_3_. Such studies show that these prepared samples are quite promising candidates for use as a tunable nanodielectric substrate in microelectronic device fabrication.

## Figures and Tables

**Table 1:** Bragg’s angle (2θ), basal spacing (d), full width at half maximum (FWHM) β, crystalline size (L), peak intensity I of the PVDF diffraction peaks of (PVDF/PMMA) with x wt% Fe_2_O_3_ films as a function of x wt%.

S.No.	x wt % Fe_2_O_3_	2θ (in °)	d (Å)	FWHM × 10^−2^ (rad)	L (nm)	I (counts)
**I**	**(100) Reflection Plane Parameters**					
1.	0	18.403	4.81	0.3070	47.755	8231
2.	0.5	18.037	4.91	0.5117	28.651	3957
3.	1	18.351	4.85	0.3070	47.776	6231
4.	2	18.299	4.84	0.8187	17.914	4401
5.	4	18.195	4.87	0.6140	23.883	4399
**II**	**(020) Reflection Plane Parameters**					
6.	0	19.807	4.477	0.4093	35.912	7935
7.	0.5	19.833	4.471	0.4861	30.239	4338
8.	1	19.937	4.448	0.3582	41.043	6802
9.	2	19.95	4.445	0.4350	33.797	4017
10.	4	19.885	4.459	0.4349	33.802	4764
**III**	**(200) Reflection Plane Parameters**					
11.	0	20.665	4.2930	0.4350	33.835	3195
12.	0.5	20.327	4.3636	0.4341	33.887	2928
13.	1	20.840	4.2573	0.3582	41.101	4069
14.	2	20.847	4.2559	0.3879	37.954	3493
15.	4	20.613	4.3037	0.3520	41.810	3494
**IV**	**(110) Reflection Plane Parameters**					
16	0	22.849	3.8873	0.5117	28.869	2652
17	0.5	22.927	3.8743	0.4093	36.096	2987
18	1	22.485	3.9494	0.5117	28.850	3370
19	2	22.745	3.9049	0.3520	41.959	3537
20.	4	22.641	3.9226	0.3897	37.893	4199
**V**	**(101) Reflection Plane Parameters**					
21.	0	24.461	3.6347	0.3520	42.091	2151
22.	0.5	24.097	3.6888	0.2047	72.329	2939
23.	1	24.201	3.6731	0.2179	67.961	6547
24.	2	24.071	3.6927	0.2047	72.326	5059
25.	4	23.941	3.7124	0.2303	64.271	6397
**VI**	**(021) Reflection Plane Parameters**					
26.	0	26.697	3.3351	0.4093	36.359	4048
27.	0.5	26.593	3.3479	0.3892	38.228	2984
28.	1	26.411	3.3706	0.3524	42.205	4044
29.	2	26.333	3.3804	0.2343	63.468	3439
30.	4	26.567	3.3511	0.2913	51.073	4683
**VII**	**(012) Reflection Plane Parameters**					
31.	0.5	30.729	2.9061	0.2989	50.239	2743
32.	1	30.441	2.9329	0.2814	53.326	3523
33.	2	30.649	2.9135	0.2980	50.381	3279
34.	4	30.623	2.9159	0.2558	58.688	4443
**VIII**	**(104) Reflection Plane Parameters**					
35.	0.5	33.041	2.7078	0.2303	65.580	4646
36.	1	33.197	2.6954	0.2184	69.182	12,035
37.	2	33.067	2.7057	0.2558	59.047	9369
38.	4	32.963	2.7140	0.2558	59.031	12,464
**IX**	**(110) Reflection Plane Parameters**					
39.	0.5	35.589	2.5196	0.2303	66.032	3728
40.	1	35.693	2.5124	0.2555	59.537	10,050
41.	2	35.511	2.5249	0.2303	66.018	6605
42.	4	35.459	2.5285	0.2558	59.428	9656
**X**	**(113) Reflection Plane Parameters**					
43.	0.5	40.790	2.2095	0.2556	60.438	2916
44.	1	40.867	2.2055	0.2535	60.954	4597
45.	2	40.789	2.2095	0.2558	60.391	4450
46.	4	40.685	2.2149	0.2558	60.371	6032
**XI**	**(024) Reflection Plane Parameters**					
47.	0.5	49.395	1.8428	0.2558	62.305	2974
48.	1	49.525	1.8383	0.2047	77.899	5168
49.	2	49.421	1.8419	0.2558	62.311	4604
50.	4	49.291	1.8465	0.2558	62.279	6554
**XII**	**(116) Reflection Plane Parameters**					
51.	0.5	53.997	1.6961	0.2558	63.529	3094
52.	1	54.023	1.6954	0.2558	63.536	4833
53.	2	53.971	1.6969	0.2558	63.521	4973
54.	4	53.867	1.6999	0.2558	63.492	6886
**XIII**	**(018) Reflection Plane Parameters**					
55.	0.5	57.51	1.6006	0.2627	62.872	2564
56.	1	57.507	1.6006	0.4097	40.313	3654
57.	2	57.481	1.6013	0.3066	53.862	3479
58.	4	57.429	1.6026	0.3521	46.890	4577
**XIV**	**(214) Reflection Plane Parameters**					
59.	0.5	62.395	1.4865	0.3719	45.516	2813
60.	1	62.421	1.4859	0.3070	55.146	4123
61.	2	62.369	1.4870	0.2558	66.166	4060
62.	4	62.239	1.4898	0.2558	66.121	5531
**XV**	**(300) Reflection Plane Parameters**					
63.	0.5	63.980	1.4534	0.5867	29.098	2786
64.	1	64.241	1.4481	0.3070	55.689	4033
65.	2	63.981	1.4534	0.2558	66.741	4039
66.	4	63.851	1.4560	0.2558	66.694	5655

## Data Availability

Data available with authors.

## Sample Availability

Samples are available from the authors.
